# The Impact of Brain Age versus Chronological Age on Cognitive Fatigue: Novel Metrics and New Insights

**DOI:** 10.1093/geroni/igaf122.4213

**Published:** 2025-12-31

**Authors:** Cristina Roman, Glenn Wylie, Jay Buckey, Nancy Chiaravalloti, James Ford, Michael Falvo, Helen Genova, John DeLuca

**Affiliations:** Keck School of Medicine of USC, Los Angeles, California, United States; Kessler Foundation, West Orange, New Jersey, United States; Dartmouth Geisel School of Medicine, Hanover, New Hampshire, United States; Kessler Foundation, East Hanover, New Jersey, United States; Dartmouth College, Hanover, New Hampshire, United States; Rutgers University, East Orange, New Jersey, United States; Kessler Foundation, East Hanover, New Jersey, United States; Kessler Foundation, West Orange, New Jersey, United States

## Abstract

Fatigue impacts 22% of the general population. Yet, our understanding of chronological age and fatigue is mixed, suggesting other more novel age- and fatigue-related metrics, such as brain predicted age difference (brain-PAD; i.e., a measure of accelerated/decelerated brain aging) and Signal Detection Theory (SDT; i.e., objective behavioral fatigue measures), are needed to better understand the cognitive fatigue (CF)-age relationship. The current study investigates the relationship between chronological age versus brain-PAD and CF in a non-medical lifespan sample (n = 85; mean age=46.9 years). Participants completed fatigue inducing tasks during brain scans, capturing real time “state” CF using a visual analog scale. Slope and SDT (Criterion) metrics were calculated for CF. Brain-PAD (i.e., chronological age minus predicted brain age) was estimated using brainageR. Linear mixed effects analyses showed a main effect of Chronological Age (F(1, 97.1)=7.33, p = 0.008): for every year of increased age, participants reported 0.51 less CF. CF slope showed a significant effect of Brain-PAD (F(1,79)=4.00, p = 0.04), such that participants with increased brain-PAD fatigued at a faster rate. There was a three-way interaction between CF, Age and Task (F(1,403.8)=5.52, p = 0.02), resulting from a positive relationship between SDT Criterion and CF when participants were young that became increasingly flat as age increased; slope became negative for older participants. Our results show a critical difference between younger and older participants in relation to CF. In addition, increased brain age, rather than chronological age, predicted faster accrual of CF. These results suggest that CF may be an effective indicator of brain age across the lifespan.

